# The feasibility and acceptability of implementing simplified cognitive behavioral therapy approaches to support postpartum mental health and address associated social and behavioral barriers to postpartum family planning in Amhara, Ethiopia: a qualitative study

**DOI:** 10.1186/s12905-025-03744-w

**Published:** 2025-05-24

**Authors:** Sarah Burgess, Tesera Bitew, Andenet Haile, Julien Souffrant, Dominick Shattuck, Lynn M. Van Lith, Jessica Moore, Zoé M. Hendrickson

**Affiliations:** 1 Gates Foundation, Seattle, WA USA; 2 Injibara University, Injibara, Ethiopia; 3 Deep Dive Research & Consulting, Addis Ababa, Ethiopia; 4 PJT Partners, New York City, NY USA; 5 Johns Hopkins Center for Communications Programs, Baltimore, MD USA; 6 Pasteur Network, Paris, France; 7 Health, Behavior and Society Department, Johns Hopkins Bloomberg School of Public Health, Baltimore, United States

**Keywords:** Mental health, Family planning, Reproductive health, Community health workers, Contraception, Maternal health, Ethiopia

## Abstract

**Background:**

Poor mental health can negatively impact health outcomes across diverse health areas, including in the first year postpartum. Yet, the intersection of postpartum mental health and postpartum family planning (FP) is understudied. Cognitive Behavioral Therapy (CBT) is an evidence-based practice that has proven helpful for improving mental health and supporting positive behavior change across health areas, including in low-resource settings. Drawing on existing CBT tools, we created and piloted an intervention called *Mothers Time,* designed to be delivered in three sessions by a community health worker (CHW) to small groups of postpartum women experiencing depression or anxiety symptoms and an unmet need for FP. Our objective was to assess the feasibility and acceptability of *Mothers Time* in rural Amhara, Ethiopia.

**Methods:**

We recruited and trained four CHWs to deliver the intervention. We recruited 16 postpartum women experiencing mild to moderate anxiety and depressive symptoms to participate. We conducted in-depth interviews with women and CHWs before, during and after the intervention. We used a framework approach to analyze data. To assess acceptability, we analyzed data from mothers, probing to understand whether they found the intervention accessible, engaging, and relevant for the challenges they were experiencing postpartum. To assess feasibility, we explored CHWs ability to deliver the intervention and analyzed their capacity to deliver it at consistent quality. Interviews with CHWs and other health actors provided additional data on the feasibility of adding simplified CBT to CHWs' current package of services.

**Results:**

Mothers perceived *Mothers Time* to be acceptable and to provide helpful tools for navigating the postpartum period. Prior to the intervention, many women felt isolated, and the group sessions supported social connection. Vignettes demonstrating simple CBT concepts engaged mothers, provoking reflection on how anxious or sad thoughts can impact behaviors that are important for informed FP use (such as care seeking, spousal communication and planning for the future) and sparked discussions on how mothers can support their own mental and physical health. Homework (explained by CHW in sessions and completed independently between sessions) helped women prioritize caring for themselves and social connection. Overall, we found that it was feasible for CHW to learn and deliver *Mothers Time*. CHW understood that mental health could create challenges for mothers and were able to use the simplified materials to share information about basic mental health concepts. Limited time was the biggest challenge; CHWs would benefit from additional training, and women would likely benefit from additional sessions.

**Conclusion:**

This research may be useful to practitioners looking to integrate mental health and postpartum FP in low-resource settings. These findings can be used as a foundation for future research and pilot interventions to support all women to meet their postpartum and FP needs, including those living with symptoms of depression and anxiety.

## Introduction

In the first year following a birth, it is common for women to experience an unmet need for family planning (FP) and mental health challenges; both can have lasting effects on maternal and child health and well-being.^1^ Unmet need for FP in the postpartum period can lead to unwanted pregnancies at shorter birth intervals, which can increase risks for adverse health outcomes for mothers and children alike [2, 3]. A recent analysis of data from 27 low- and middle-income countries estimated that about 65% of all women 0–12 months postpartum had an unmet need for FP [4]. Across contexts, women who recently gave birth commonly express a desire to wait until achieving another pregnancy [5]. Among postpartum women with unmet need, commonly cited reasons for non-use include that their menses have not started, concerns about side effects, and lack of spousal approval [6]. Lack of access to services, along with lack of quality counseling, may also be barriers to postpartum FP [6].

Also following a birth, it is common for women to experience depression and anxiety.^2^ In a systematic review on data from low- and lower-middle-income countries, the weighted mean prevalence of women experiencing common mental disorders (including anxiety and depression) postpartum was 19.8%, or about 1 in 5 [9]. Poor postpartum mental health negatively affects mothers and can lead to negative health impacts for infants and children. Postpartum mothers with depressive symptoms have been shown, for example, to be more likely to discontinue breastfeeding, report lower levels of breastfeeding self-efficacy, bottle feed, and engage in early weaning [10]. Poor maternal mental health can also be detrimental to the physical and neurological development of infants and children [11].

Despite the high burden of these two challenges after birth, there remains a paucity of research on the intersection of postpartum mental health disorders (such as depression and anxiety) and FP behavior to understand how mental health influences fertility intentions, decisions, and practices and vice versa.^3^ There is also a lack of interventions and tools for postpartum FP that consider the needs of those struggling with mental health. As a result, there is an urgent need to understand how best to serve women during the postpartum period to address both reproductive and mental health concerns.

Though the literature looking specifically at the intersection of mental health and FP is limited, there are lessons that can be drawn from other health areas for how mental health affects individuals’ capacity for engaging in behaviors that protect and promote their own health. For example, mental health difficulties can negatively affect Ebola risk behavior [12]; increase the risk for malaria [13]; erode treatment adherence for people living with HIV/AIDS [14]; and reduce communication with healthcare workers [15]. Researchers have provided various explanations for how exactly mental health affects health behavior and outcomes, positing for example that mental health conditions can: cause a sense of hopelessness that increases the likelihood of risky behavior [12]; decrease preventative behavior [13]; and disturb concentration, sleep, and energy and thus disrupt motivation and self-efficacy [14].

These same mechanisms may also link postpartum mental health and women’s reproductive health behaviors, including contraceptive use. While the research on mental health and reproductive health is limited, available studies are generally consistent with those focused on other health areas that show that mental health conditions like depression and anxiety can make it more difficult for individuals to plan and take action to promote their current and future health. Studies from the United States have shown that women with depression may be more likely to choose less effective FP methods [16], use methods less consistently [17], or discontinue oral contraceptives [18]. Findings from a recent systematic review and meta-analysis suggested that women with psychiatric vulnerabilities have higher risk of unintended pregnancies [19]. A recent study in Ethiopia found an association between mental health symptoms post pregnancy and unmet need for FP later [20]. Although there is limited research on the mechanisms that link poor mental health with low FP uptake, one Ethiopian study posited that poor mental health may impact women’s social connections, limiting their awareness of health messages and inhibiting their ability or motivation to advocate for the FP services they need [20]. In the US, research found that women with a depressed mood (or stress) may experience contraceptive side effects more intensely than others, leading to increased discontinuation [18].

Multiple social and structural factors can be barriers to FP use [21]. It follows that women experiencing symptoms of depression and anxiety may have additional challenges in navigating these barriers. Studies at the intersection of mental and reproductive health call for integration of these two health areas, and development of tools for FP counseling and education that support all women, including those living with anxiety and depression.

However, there remains a lack of available tools that are specifically designed for this purpose, in part because mental health services throughout the world are nascent and few providers have specialized training in mental health [22]. In many low- and middle-income countries (LMICs), poor mental health and mental health conditions continue to be stigmatized, with social norms making it challenging to discuss openly [23]. The combination of these factors means that any intervention designed to address mental health must be designed and tested with attention to local context and resource constraints.

This paper describes the feasibility and acceptability of implementing a locally contextualized intervention called *Mothers Time* designed to address the gap between mental health challenges and postpartum FP use. To design the intervention, we reviewed literature on interventions designed for low-resource settings, drawing heavily from interventions using simplified Cognitive Behavioral Therapy (CBT). CBT is often considered to be the gold standard in the field of psychological treatments [24].^4^ While research on mental health approaches in LMICs is limited, an emerging body of evidence documents how CBT programs in low-resource settings have been used to simultaneously address mental health and support positive outcomes in other areas, including, for example, trauma in Zambia [27], HIV risk behaviors in Uganda [28], and exclusive breastfeeding in Pakistan [29]. Perhaps the most widely known and widely used CBT intervention specifically for postpartum mothers is called *Thinking Healthy*, which has shown to reduce depression and improve maternal and infant health outcomes, including contraceptive use [30]. Thinking Healthy was initially developed for use in Pakistan and has been adapted and implemented in several countries and low-resource settings, including India, Bangladesh, Peru, Vietnam, Bolivia, and Nigeria. Although the Thinking Healthy module available through WHO for adaption in other settings briefly touches on child spacing, it has limited content on FP [31].

Before designing a study to assess the impact of *Mothers Time*, we aimed to determine if such an intervention was feasible to deliver and acceptable to the population it aimed to serve. This paper describes the process of designing and assessing the feasibility and acceptability of *Mothers Time*. Below, we describe how the intervention was adapted from existing CBT tools and practice materials and methods for recruiting participants and collecting and analyzing data. To assess acceptability, we collected qualitative data from mothers before, during and after the intervention, probing to understand whether they found the intervention accessible, engaging, and relevant for the challenges they were experiencing postpartum. To assess feasibility, we collected qualitative data from community health workers (CHWs) and analyzed their ability to deliver the intervention and their capacity to deliver it at consistent quality. Findings may be used to inform future interventions at the intersection of postpartum mental and reproductive health.

## Methods

### Setting

This study was implemented in Ethiopia based on its existing commitments and infrastructure. Ethiopia has existing national coordination, infrastructure, and investment for both FP^5^ and mental health.^6^ This study was implemented in Awi Zone of the Amhara Regional State, a rural area in northwestern Ethiopia with a population of 982,942 [37]. Awi zone is a flat, fertile region, sitting about 114 km away from Bahir Dar, the capital of Amhara, and 449 km from Addis Ababa [37]. The area is largely rural, and access to services and infrastructure is limited. In a study on women who had given birth in Awi zone, most women (92%) were orthodox Christian; 55% had not completed any primary education [38]. Only 75% had access to clean drinking water, 62% toilet facilities, and 79% had made an antenatal visit during their last pregnancy [38].

Awi Zone has five government hospitals and 46 health centers; meaning each health center serves about five health posts [39]. Health posts are grassroots level healthcare facilities providing services such as health promotion and illness prevention. Each health post is staffed by community-based Health Extension Workers (HEWs), who are Ethiopia’s CHWs. HEWs deliver a package of prevention-based care, including hygiene and sanitation; disease prevention and control; family health (including maternal and child health and FP); and health education and communication [40].

### Intervention development

Working with a postpartum mental health specialist (a co-author), we developed a three-session intervention for postpartum women called *Mothers Time,* designed to be delivered by HEWs to postpartum women experiencing mild to moderate symptoms of depression or anxiety as well as having an unmet need for FP. To develop this guide, we drew heavily on the theory and materials from *Thinking Healthy* [31] as well as the materials and approaches from other simplified CBT interventions designed for postpartum women such as those from *Mothers and Babies* [41]*.*

*Mothers Time* is designed to be delivered by an HEW to a small group of four to six women. Other CBT models (such as the WHO version of *Thinking Healthy)* for low-resource settings are designed to be delivered in 1-on-1 sessions between a woman and a CHW [30]. The decision to deliver the intervention in group sessions (as opposed to individually) was made to reach a greater number of women and also in recognition that the group itself could be supportive for the women involved as they could share experiences and learn from one another. Consultations with local stakeholders also informed this decision; HEWs in Ethiopia have intense workloads and it would likely be difficult for them to find time to deliver individual sessions to women, both in our study and in future iterations of the intervention.

The version of *Mothers Time* tested in this study was comprised of three sessions, each focusing on a different theme. The sessions included explanations of CBT concepts and methods in simplified language. For example, “unproductive thoughts” were explained as “thoughts that are not helpful” for a person that can take away their focus from goals or prevent one from feeling empowered. “Productive thoughts” were defined as thoughts that are helpful for a person and their health. HEWs led discussion about types of unproductive thoughts (such as “not believing in oneself”) and “alternative thoughts” that can be more helpful for a woman’s well-being and health. Group discussions and homework completed outside group time prompted women to notice types of activities that helped them in having alternative, helpful thoughts (such as spending time with others, relaxing) and prompted them to intentionally make time for those activities.

Given the low access to education in rural Amhara, all content was designed to work with mothers with no literacy skills. We also used language designed to be non-stigmatizing; for instance, we used terms like “sadness” and “stress” for depression and anxiety. Much of the intervention utilizes fictional vignettes centered around the experiences of Birhan, a young woman who is struggling with sad and anxious thoughts as she navigates life with a new baby. Vignettes were complemented with illustrations of Birhan that were created by a local artist. The vignettes did not explicitly focus on FP, but included examples of anxious or depressive thoughts that may influence FP decision making. Intervention materials guided the HEW in leading discussions about Birhan’s thoughts, how those thoughts might impact her behavior, and what might happen if she tries changing her thoughts. Participants were also invited (but never required) to share and discuss their own challenges and unhelpful thoughts. Sample content is included in Table 1; a summary of the content in each intervention session is included in Table 2.

**Table 1:** Sample content^a^

Example: One of Birhan’s “unproductive thoughts” discussed in session 3	Example: One of Birhan’s “helpful thoughts” discussed in session 3
I’m overwhelmed taking care of my baby. I don’t have time to think about the future or my health. It doesn’t matter what I do.	It is a lot of work to care for my baby right now. But it’s important that I make time to think about my health and my baby’s health. The choices I make matter.

^a^The images in this table were developed for this study, as part of the Breakthrough ACTION project

**Table 2:** *Mothers Time *content

**Session**	**Content**
1. Introduction to healthy thoughts	• Introduction to the intervention and norms for group discussion together (such as respecting confidentiality) • Introduction to the character of Birhan • Introduction of basic CBT concepts (helpful and unproductive thoughts; types of unproductive thoughts; activities that help challenge unproductive thoughts) • Introduction to homework
2. Thinking healthy about yourself	• Review of homework • Vignette: Birhan’s thoughts about the future • Vignette: Birhan’s thoughts about communicating with her husband
3. Thinking healthy about your family	• Review of homework • Vignette: Birhan’s thoughts about visiting a health worker • Vignette: Birhan’s thoughts about a friend’s experiences with side effects • Exercise: Reflecting on your support network

### Participants and recruitment

Participants included individuals involved with the intervention (16 mothers participating in the implementation of the intervention and four HEWs implementing it) as well as six other actors in the Ethiopian health system knowledgeable about mental health and FP. These six other actors included three health promotion volunteers, also known as members of the Health Development Army, and three midwives. Recruitment began in November 2021.

The study team used a purposive sampling approach. First, working through contacts at the Ministry of Health, the study team recruited HEWs to facilitate three *Mothers Time* groups. The study team screened to recruit HEWs who regularly provided care to postpartum women, regularly delivered FP services, and were 18 years or older. Eight HEWs were screened, and all eight were eligible and consented to participate. All eight HEWs were trained to deliver *Mothers Time;* two dropped out due to scheduling constraints, and four fully participated in the study. (The two remaining HEWs served as alternates, in case of additional drop-outs.)

Once HEWs were recruited, we then worked within their catchment areas to screen and recruit 16 mothers ages 16–25 who were married, had given birth in the last year, were not currently using a method of FP, and reported mild or moderate symptoms of depression or anxiety. The Patient Health Questionnaire-9 (PHQ-9) and Generalized Anxiety Disorder-7 (GAD-7) were used to identify people who report symptoms of depression and anxiety, respectively.^7^ These screeners are widely studied and used globally by mental health practitioners [42, 43].

We used versions of these screeners that had already been tested in the Ethiopian context; the PHQ-9 had been statistically validated [44] and the GAD-7 had been adapted for local context and piloted [45]. We screened 63 women for participation; 16 were eligible and all 16 consented and enrolled.

To qualify for the study, participants needed to score between a 5 and 14 on the PHQ-9 screener and/or between 5 and 14 on the GAD-7 screener. On both screeners, “5” is a cut off point for mild depression or anxiety [42, 43]. On the PHQ-9, a score of 15 indicates “moderately severe” depression, and on the GAD-7, a 15 indicates “severe anxiety” [42, 43]. As the intervention was not designed to meet the needs of women with high levels or depression or anxiety, we established a protocol to refer them to other services.

Other health care actors in our sample (the three midwives and three health promotion volunteers) were also 18 or older and had provided FP education or promotion in the last 12 months. These participants were not involved in piloting *Mothers Time* but were included to provide additional perspective on FP and postpartum care in Amhara. We screened 9 other health care actors, 6 were eligible and enrolled.

### Data collection and analysis

Data collection was primarily qualitative. We conducted three rounds of in-depth interviews with HEWs and mothers (one before the intervention, one after the second session, and one at completion of the intervention). Interviews included open-ended questions and lasted about 60 min. Interviewers were trained to probe and slightly adapt the interview according to the unique circumstances of each participant. The introductory interview included questions on the process of being recruited for the study and intervention participation, anticipating that this data would be helpful for designing future recruitment approaches for postpartum mental health. The second and third interviews focused on the content of the intervention and participants’ perspectives and reflections. The final interview with mothers, completed after the final *Mothers Time* session, also included the PHQ-9 and GAD-7 questionnaires to screen participants again for depressive and anxiety symptoms.^8^ Two mothers were unavailable to complete the midpoint interview; all other interviews were completed as planned.

To complement interviews, we conducted structured observations at the HEW training and *Mothers Time* group sessions. Research assistants attended both the training and the sessions and took notes using structured templates to collect data on the extent to which mothers and HEWs understood and engaged with the content. Separately from the *Mothers Time* pilot, we also conducted six single in-depth interviews with the other health actors (three midwives, and three health promotion volunteers).

All interviews were audio recorded and observation notes were taken by hand. Interviews were conducted in Amharic and the word-for-word transcriptions were translated into English.

Data were analyzed by multiple team members, using an approach which centered around participants’ journeys throughout the intervention, drawing on approaches used in other qualitative studies aiming to understand change over time [46, 47]. We created analytic frameworks for each type of participant (mothers, HEWs, and other health actors), and organized data from each participant by key categories such as FP, mental health, and social context. Appendix 1 includes a summary of the key themes and sub-themes used for analysis among each group of participants. We entered the data into the framework using color coding, which allowed us to track how participants’ experiences evolved over time. (Note that “Other Health Actors” were only interviewed once, and thus not followed over time.)

We then reviewed data coded to each category and synthesized it to surface key patterns that occurred across multiple participants and were relevant to study objectives. We then grouped data from participants who shared similar characteristics (such as a common barrier to FP use) and identified commonalities and differences within these groups. Each team member of the analysis team focused on a specific set of participants; team members also compared and reviewed each other’s approaches to ensure consistency and verify analytical findings. Finally, we also looked at screener scores from the PHQ-9 and GAD-7 and compared mean scores from baseline to endline.

### Ethics

The research received ethical approval from the Amhara Public Health Institute Regional Public Health Research Ethics Review Committee in Ethiopia and the Johns Hopkins School of Public Health Institutional Review Board (IRB) in the United States. All participants were briefed on the purposes and procedures of the study, provided oral informed consent prior to participation, and were given the option to stop participation at any time. Per guidance from both IRBs, mothers who were ages 16–17 but married were considered emancipated minors and thus had the same screening and informed consent procedures as mothers age 18 or above.

In the screening process and throughout the interview, referral protocols were established to ensure that participants who showed severe symptoms of depression or anxiety (scoring 15 or higher on either the PHQ-9 or GAD-7, or reporting suicidal ideation) were advised that the intervention would likely not be helpful for them and were given resources to visit psychiatric services, if desired. However, in the study procedures we did not identify women who met the criteria for referral. The study team and all participants took measures to prevent the spread of COVID- 19, including daily screening, mask wearing, and use of disinfectants.

## Results

In this section, we describe data providing context on mothers and HEW participants and the feasibility and acceptability of *Mothers Time*. Findings are presented separately for mothers and HEWs. The first subsection of this section provides context on mothers' lives before the intervention. The second section (exploring mothers’ reactions to different components of the intervention) and third section (exploring changes that mothers experienced during the intervention) shed light on acceptability. The fourth subsection provides context on HEWs and their approach to providing care to postpartum women (prior to the study). The fifth shares results on data that speak to the feasibility of HEWs learning and delivering *Mothers Time.*

### Mothers’ lives before the intervention

Per inclusion criteria, the sixteen mothers enrolled in this study were married, had given birth to a child within the last year, and lived in a rural area of Amhara. Women scored between 1–11 for the PHQ-9 (mean of 8.8) and 0–7 for the GAD-7 (mean of 4.7), reflecting mild to moderate symptoms of anxiety and/or depression. While our screening process recruited women between the ages of 16–25, participants were between 19–25. Mothers had varied levels of education, family size, and relationship dynamics. While some had little or no literacy and limited schooling, others had been to college. Some were new to motherhood and raising their first child; one woman had three other children in addition to her new baby. Most women were juggling multiple responsibilities, including childcare, household work, agricultural responsibilities, and other work. They had varying strategies for coping with life challenges; one mother described sleeping for just six hours and working hard all day long. Mothers had concerns about health challenges, financial stability, and political conflict in Ethiopia.

### Social relationships

Women described varied relationships with their husbands; some had experienced long courtships and chose their partners, while others had arranged marriages. Husbands had varied occupations, including farmer, laborer, and teacher. Several women’s husbands had multiple jobs, and some had migrated to other parts of the country to work. The level of communication and shared decision-making varied among couples, with some describing deep connection and shared decision-making and others explaining that they felt some distance from their spouses. Communication was particularly challenging for those whose husbands had gone elsewhere for work. “Sometimes we talk on the phone about different things” said one mother, citing recent examples on their child and household financials, “but it is not a real discussion.”

Social isolation was a common theme across interviews. While some women described close social connection with friends and family, many were separated from their families following their marriage, separated from their usual routine or network following the birth of their child, or lonely while their husbands engaged in migrant work elsewhere. “Life after giving birth is different” one woman noted, “my movement is restricted, I cannot be as I want to be, I cannot do what I love to do.” Another noted that her recent marriage had caused a rift with her family, saying, “I feel like I disgraced my family. They didn’t approve of my marriage at first, but I insisted on getting married. Whenever there is a misunderstanding between me and my husband, I blame myself and I feel like I disgraced them.”

### Family planning

Women in the sample were generally aware of FP. Most wanted to delay their next pregnancy by three years or more. In general, women’s primary reasons for non-use fell into three categories. The first group was not using due to infrequent sex or their husband’s absence from the household. Although some of these women were not experiencing an immediate need for FP, there was often ambiguity around when their husbands would return, suggesting they may have need in the very near future. Another subgroup of women was concerned about side effects; two within this group also cited opposition from family and/or religion. The third group was interested in using contraception but had not yet obtained a method, explaining they were busy with other priorities.

### Mental health

In introductory interviews, women shared details of experiences or symptoms that are consistent with the clinical definitions of depression and anxiety. “Sometimes I feel a load of tension” said one mother, “I can’t sit still, I will be irritated, I feel hopeless, and I will be tired without working.” Another described emotional changes which occurred after her recent birth. “After giving birth” she noted, “my attention is distracted, I get annoyed easily, everything and everyone irritates me, I don’t enjoy things as I used to, and I get tired easily.” One mother noted, “when I am stressed, I usually get sick.” One participant described her own coping mechanism for dealing with pain, stress, and anxiety: “When I have these feelings, I pour water on my head and wash my face, which gives me some relief.”

## Mothers’ experiences: acceptability of intervention components

### Recruitment

Over the course of the study, we collected data from mothers on their experience with recruitment, and different components of the intervention. The study team was aware that the recruitment experience may be stigmatizing, as both mental health and FP can be sensitive topics. In the recruitment process, therefore, both the study team and the HEWs who had contact with potential participants used simple, non-stigmatizing language to describe the intervention. Overall, stigma did not emerge as a key challenge with recruitment. Several women recognized that they were facing mental and emotional challenges and expressed appreciation for an opportunity for additional support. “I like the fact that they considered me” noted one woman, “because they know I had difficulties.” One noted that she was hesitant to agree to participate during recruitment because of her limited time, but later was glad she signed up to engage. Multiple participants suggested that future iterations of the activity reach more women, instead of including just those who met the screening criteria for inclusion.

### Intervention content

Interviews at midline and endline probed into whether women found the content of the intervention acceptable and relevant. In interviews, women tended to focus on the fictional character used throughout the intervention, named Birhan. Mothers reported that they related to the character of Birhan, which seemed to support their engagement and participation. “I saw myself in Birhan’s story” noted one mother. “Birhan was a character to show us our struggles, thoughts and emotions” noted another. When asked to recall key lessons from the sessions, participants often recalled specific details about Birhan’s stories.

In interviews, the study team asked participants to explain key concepts taught to assess their understanding of these concepts. Some participants were able to provide detailed and accurate definitions of these terms. “Unproductive thoughts” explained one participant, “are demotivating and negative.” She went on to provide several examples, noting that, “these thoughts disable the person from taking action and feeling good.” The same participant explained that healthy thoughts enable people to “discuss with others and address one’s own needs.” Other participants provided definitions that were less detailed or anchored to specific examples from the Birhan story.

### Homework

Overall, women had positive experiences with the homework, reporting that it helped them make time for themselves, prioritize themselves, and start conversations with their spouses.

Several women in the cohort reported feeling isolation before the sessions, and the homework encouraged them to make time for social connections. One woman noted, “If it was not for the homework, I would not have talked with both my friend and my husband. I gave attention to those activities because it was homework. I was happy after meeting with my friend and talking with my husband.” A few women also provided feedback and suggestions on design modifications to make homework more accessible for women with low literacy.

### Intervention format

Many mothers had described feeling isolated, and delivering the intervention through a group setting (as opposed to individual 1-on-1 sessions) provided opportunities for social connection. One woman noted that prior to the intervention, she was “just sitting at home doing nothing” and without social contact, she had the tendency to “overthink by myself.” She expressed appreciation for the encouragement to connect with others that came through the program.

Interviews also solicited women’s opinions about their preferences for the type of health actor who could deliver the intervention (HEW, midwife, volunteer, or other). In parallel, interviews with midwives and health volunteers probed into their interest, capacity, and availability to deliver an intervention. Input from mothers suggested that HEWs were the best positioned to deliver a simplified CBT intervention, having the best balance of time, community relationships, and expertise.

## Mothers’ experiences with changes after the intervention

### Shifts in mental health

In evaluating acceptability, we collected data to understand if mothers experienced the intervention as potentially useful to navigating challenges relating to mental health and FP. Overall, participants reported positive experiences, noting that it was helpful addressing emotions like stress and sadness. A comparison of scores for depression and anxiety at baseline and endline showed that PHQ-9 scores dropped from 8.8 to 2.6, and GAD-7 scores dropped from 4.7 to 2.3, substantial changes. In interviews, women reported that the intervention had positive impacts on their mental health. One woman noted that the most significant change she experienced was learning to “turn non-constructive ideas into constructive ideas.” While these results are promising, this sample was small, and data may have been impacted by social desirability bias. The screening data on anxiety and depressive symptoms was collected immediately after the intervention, and thus cannot be interpreted as an indication of long-term change.

Another woman noted that participation in the program offered an opportunity for her to speak with her husband about the challenges she was experiencing. She was able to explain to him that her struggles were “serious” and reported that after this conversation, he began to help more around the house, giving her more time to care for herself and for her baby. The group setting also provided opportunities for HEWs to connect with mothers and refer them to other services as needed. Other women, however, reported that by focusing only on mothers, the program had limited impact. Speaking about her relationship with her husband, and whether it had changed, one woman noted, “nothing has changed between us, except my emotion.”

### Shifts in FP

Overall, participants also reported that the intervention had given them more time and space to consider their FP intentions. As discussed earlier, prior to the intervention, women cited three reasons for not using FP. The first group’s primary reason for non-use was that their husbands were away, or they had not yet resumed regular sex. The second group’s primary reason for non-use was side effects and opposition from religion and others. The third group described delays in FP decision-making or care-seeking. Women in these three groups described subtle changes stemming from the intervention.

Prior to the intervention, most mothers in the first group noted that they planned to begin use once their husbands returned, or once regular sex resumed. It was not clear, however, if women in this group were able to communicate with their partners about when sex might resume or their FP needs. After the intervention, women in this group described subtle changes, such as improved communication with spouses or a better sense of which specific method they might use in the future. One mother in this group, for example, explained prior to the intervention that she was not using FP because she has not yet resumed sex with her husband, and also had concerns about side effects. She also described feeling overwhelmed with responsibilities and worries. She reported that the intervention motivated her to communicate with her husband, who had sympathy for her and agreed to take on more household responsibilities. She then had more time to care for herself and described increased motivation to visit a health care worker.

After the intervention, women in the second group – those most worried about side effects or opposition from others – generally expressed new confidence in navigating side effects. One participant, for instance, described multiple anxieties prior to the intervention, including stress about her household responsibilities and the political situation in Ethiopia. Concerns about side effects were the primary reason she was not using contraception. In the final interview, she noted that one of her key takeaways from the intervention was that it may be unproductive to worry about side effects without consulting health workers, who may be able to help with her concerns.

At the end of the intervention, women in the third group – who were not using due to delays in seeking a method – shared that they were more motivated to seek support from health professionals or others in their communities. One mother in this group noted that she wanted to use FP but had not yet made time to obtain a method. Later, in interviews, she noted that she and fellow mothers had a tendency to keep problems to themselves, but following the intervention, she realized this behavior “is not good for us, and we should discuss with health workers if we have issues or need help.”

Though most participants reported some nuanced change around their FP considerations or behavior, one participant in the final interview described religious barriers which were not addressed in the intervention, saying, “I did not consider family planning using the modern methods since God prohibited that in my religion."

## Health extension worker roles prior to *Mothers Time *

### Workload and responsibilities

In the first interview, prior to *Mothers Time* training, HEWs were asked to describe their current workload or responsibilities. They described long days, busy schedules, including lots of engagement with pregnant women and women who recently delivered. “I provide services like vaccines, family planning, monitoring, first aid and health education” said one HEW, explaining that, “the program is vast and huge and has many activities within it.” One key responsibility for HEWs is engaging with women at multiple key touchpoints before and after a birth, providing services and counseling on breastfeeding, vaccines, infant health, FP, and more. “We see a mother seven or eight times” one HEW explained, noting that through this long-term engagement, “we can establish a strong relationship.”

### Current approaches to mental health and FP

HEWs provided details about their current approach to postpartum FP, noting that FP counseling typically happens at 42 days postpartum. HEWs described meeting mothers 1-on-1 and using visual aids to discuss different methods. “We show them the picture of different family planning options like Depo for three months, pills for 28 days, or the implant for three years” one HEW explained. HEWs shared their perceptions on why some postpartum mothers might not use, noting that they typically want to space their children but have concerns about religious or spousal opposition or side effects.

Basic mental health was recently integrated into the package of care offered by HEWs [48], but at the time this intervention was delivered, it appeared that participant HEWs did not all use common or standardized tools to address postpartum mental health. However, participant HEWs were aware of maternal mental health challenges and spoke about their impacts on mothers and infants. One HEW observed, “if a mother is not at peace…she cannot care about her health or her child’s health.” Another noted, “most importantly, mothers feel depressed if they are alone.” HEWs described varying approaches for supporting mothers in their communities who seemed to be struggling emotionally. “The only thing we can do” one HEW explained, “is provide advice and make them feel strong.” Another noted, “I may spend more time on an emotionally stressed mother than a healthier one.” In a case where a mother had been abandoned by her husband, one HEW described collecting funds to support her.

Overall, HEW were already well immersed in postpartum mental health and FP through their daily work, but had more tools and training specifically for FP.

## Feasibility: health extension worker experiences in training and delivering the intervention

To prepare to deliver the *Mothers Time* intervention, HEWs received two days of training plus ad hoc coaching throughout the intervention. Local Ministry of Health representatives advised that HEWs had large workloads and would not be able to take more than two days of time away from their regular duties. In training, HEWs received background on postpartum mental health (specifically on anxiety and depression), CBT, and FP. Most of the training time involved engaging in role plays with the intervention content.

### Feasibility of training

In training, many mental health concepts seemed new to HEWs. HEWs asked for clarification on several terms, and the trainer provided examples to facilitate understanding. HEWs shared their own examples of encountering women with postpartum mental health challenges, suggesting they understood basic concepts and the content resonated with their current experiences. In interviews, HEWs described the training as engaging and relevant. “The contents were good” one HEW noted. “The training was delivered with examples, and simple language we can understand.” HEWs appreciated the opportunity to learn more about mental health, with one noting, “I didn’t have good knowledge about mental health before, but now I’ve had good lessons from the training.” Although the HEWs developed some basic competencies through training, the HEWs and the trainer noted that two days were not enough for full mastery. Thus, the trainer provided coaching and supervision throughout implementation.

### Comprehension of content and implementation of the approach

Following training, HEWs were tasked with delivering the intervention to groups of four to six women. Using positive reinforcement helped HEWs connect with mothers and teach them the CBT concepts. One HEW, for instance, provided mothers with encouragement during the sessions, (saying, “good job” and “you mothers are doing great”) which seemed to support mothers’ confidence and engagement. Several mothers brought their babies with them to the session, and HEWs adapted or managed the group when a baby needed attention from a mother. Overall, HEW’s comfort with the material improved as they gained more experience delivering the sessions. HEWs seemed to perform best when they were able to use examples from Birhan’s story to explain mental health concepts.

## Discussion

Overall, this study found that the *Mothers Time* intervention is acceptable to women and feasible to deliver. It may present a novel approach for supporting women during the critical postpartum period. Results also provide some limited insights into the potential ways that mental health symptoms can impact FP behavior and additional considerations for practitioners looking to design programs to meet their needs.

Data show that the intervention was feasible for HEWs to deliver. Mental health was not a completely new concept to HEWs; they had already noted the challenges it created for mothers in their community and were interested in learning new ways to support women experiencing anxiety and depression. HEWs were able to learn *Mothers Time*, and deliver it, however, the workload and burden on HEWs, and the limited scope of resources for mental health, created the biggest challenges for feasibility. Given the scope of HEW’s duties, we were only able to provide them with two days of training at the beginning of the intervention. This limited time was not enough time for HEWs to deliver the intervention at consistent quality, and they needed additional coaching to support their performance. Developing new mental health systems in other contexts will likely require expanding mental health capacity of providers who do not specialize in mental health [22]. The challenge our study experienced here – limited time – is consistent with other initiatives which have aimed to shift tasks to CHWs to expand access [49].

Data also showed that the intervention was acceptable to the population it intended to serve. Mothers connected with the stories in *Mothers Time* and used them to talk about challenging topics such as mental health, relationships, and FP. Prior to the intervention, many mothers felt isolated as they navigated the transition of new motherhood. Birhan’s stories allowed participants to explore specific examples of how unproductive thoughts can negatively impact one’s relationships, future plans, or engagement with health workers as well as how identifying unhelpful thoughts, and replacing them with helpful thoughts, can be helpful for oneself and one’s family. This suggests that using stories or short narratives was engaging, and supported participants in connecting with the intervention and discussing topics relating to mental health. Group sessions supported social connection and exchanges on strategies for addressing shared challenges. Homework helped women prioritize caring for themselves and connecting with others. Women described how the intervention was helpful for supporting them to solve challenges related to their wellbeing, relationships, and health. For instance, women who were feeling isolated expressed new motivation to connect with others and make time to care for themselves. Women reported taking actions such as increasing communication with their spouses or making plans to visit a health facility to learn more about FP.

Although this study was not designed to measure impact, results provide some insights into how mental health can negatively impact behaviors and capacities that are important for informed decision making in FP. In recent years, the reproductive health field has developed new ways of understanding FP choices and behaviors, with developed literature on social norms [50], social networks [51], gender [45], and behavioral/environmental factors [52] that can be drivers and barriers of FP decision-making and use. Yet, relatively little attention has been paid to common mental health disorders, such as depression and anxiety, and their potential effects on behaviors that are of key importance for FP – such as couple communication, care-seeking, and future planning. Common global reasons for non-use of contraception, however, appear to overlap with, or share much in common with, symptoms of common mental disorders of anxiety or depression.

The links and overlaps observed in this study are consistent with the explanations of previous authors who have sought to understand potential links between mental health and unmet need for FP [20]. Women in this study reported isolation and social withdrawal, which may have made them less likely to communicate with their husbands and connect with health workers to discuss their needs. One group expressed acute concerns about side effects, which may have been aggravated by sadness or stress. Another group of women in the intervention reported that while they were interested in obtaining a method of contraception, they had not yet been able to prioritize doing so, which could potentially reflect diminished motivation or capacity caused by depression or anxiety. Although this study is small, and it is not possible to directly attribute changes in FP intentions with improved mental health, there are important connections between mental health, couple communication, and FP that deserve further exploration.

Results provide additional insights for practitioners aiming to reach women with postpartum FP services. Our findings suggest that women experiencing postpartum depression or anxiety may be struggling with isolation, and that interventions may need to come to their communities rather than expecting them to make visits to health facilities. While privacy around mental health and FP are important considerations, we also saw the value of providing group connections and opportunity for social exchange to women who were otherwise isolated. The structured approach of simplified CBT, which prompts women to consider the links between certain thoughts, feelings, and actions, was, in this study, acceptable to mothers and supported them in exploring both mental health and areas important for FP use, such as couple communication.

This study also contributes to the growing literature on delivering mental health interventions in LMICs. Health systems around the world suffer from a dire lack of trained mental health specialists; evidence has shown that this shortage can be addressed, in part, by training non-specialist health care providers (such as CHWs or lay workers) to provide basic screening, diagnosis, treatment, and monitoring to individuals with mental health disorders [53]. Though this approach can be effective, task shifting can generate new challenges; CHWs can become overburdened [54]. Likewise, in this study context, local health officials advised that HEWs had numerous responsibilities and limited time to lead additional new interventions, especially those that would require delivery through multiple sessions. To navigate these challenges, *Mothers Time* was developed to be much shorter than many existing CBT interventions, including just three sessions. HEWs were trained over two days, during times which did not conflict with their other responsibilities. Women would likely benefit from additional sessions, which would allow them more time to explore their own specific challenges, beyond those in Birhan’s stories. Yet, we also observed through this study that a shorter intervention is still acceptable to participants with mild or moderate depressive or anxiety symptoms and may have offered some benefits. In areas where resources are limited, there still may be benefits to offering shorter interventions, though referral mechanisms must be in place to support participants with serious mental health needs. Developing short-term mental health interventions could also be an important step for countries working to build and develop comprehensive mental health services.

An intervention at the intersection of mental health and FP raises important questions about reproductive choice and informed consent. Recent scholarship on informed consent has highlighted that contraceptive coercion (which can include both forced use of FP methods and denial of FP methods) does not necessarily manifest in a single event or interaction but can occur through systems where power imbalances are present [55]. The troubling history of reproductive coercion towards women with mental disorders may make practitioners hesitate to design FP services specifically for women struggling with anxiety or depression. The data from this study, however, suggest that women struggling with anxiety or depression may need specific support and resources to make full and informed choices, and thus full commitment to reproductive choice requires us to consider these needs seriously. More work is needed to understand how mental health can impact fertility behavior, and the types of programs and policies which can support all women with access to reproductive health services, should they desire to use them.

## Limitations

This research has several limitations. Our sample was small and limited to a specific region of Ethiopia. Due to the limited scope of the study as well as the study’s focus on exploring feasibility and acceptability of the intervention, we were unable to include a control group to examine differences in mental health and FP outcomes across participants. These choices were intentional for the objectives of this study (which focused on feasibility and acceptability), but this approach prevented us from analyzing effectiveness. To address this gap, a follow-on study was planned to evaluate the impact of the intervention on both mental health and FP outcomes.

Second, this study focused specifically on women with mild to moderate symptoms of depression and anxiety, and its findings should not be used to inform interventions for women with severe depression, anxiety, or other mental health conditions.

Mental health and FP can be sensitive topics, and so interviewers took care to help participants feel at ease and ensure confidentiality. However, participants could have still limited the information they shared about these topics. Although participants offered both positive and critical feedback on the intervention, social desirability bias may have motivated women to try and please the interviewers, leading them to emphasize positive aspects of their own experiences. Data took place during the COVID- 19 pandemic, and during the Tigray conflict, which both undoubtedly shaped the mental health and well-being of participants. Although we discussed these topics in interviews, we did not probe deeply on them, and are thus not able to share insight on how they might have affected participants’ experiences with the intervention.

## Conclusion

While literature on the determinants that can affect FP behavior exists, less attention is paid to how mental health challenges such as anxiety and depression affect contraceptive choices and decision-making. While these mental health challenges are often stigmatized across cultures, they are very common and likely influence women with unmet need for FP, particularly in the first year after birth. In this study, we examined the feasibility and acceptability of using *Mothers Time* to address depressive or anxiety symptoms which can be barriers to FP uptake. Our findings shed light on how HEWs and other CHWs can be trained to deliver simplified CBT, and how mothers respond to and engage with simplified CBT and vignettes. Though this study was not focused on impact of the intervention, data also highlight how depression and anxiety can negatively affect women’s capacity for health-seeking behavior around FP and how mothers described the changes as a result of participation in the intervention.

This research may be useful to practitioners exploring the integration of mental health and postpartum FP in low-resource settings. This research contributes to the gap in the literature at the intersection of FP and mental health. This study was small, and we believe more work needs to be done to understand how mental health impacts FP, particularly in the postpartum period. This study can be used as a foundation upon which to conduct additional research to better understand how to best support all women in meeting their reproductive health intentions and incorporate mental health into community health systems.

## Data Availability

The datasets generated and/or analyzed during the current study are not publicly available due to confidentiality of the participants. High-level descriptive statistics and coded segments of text are available upon reasonable request. To make a request, please reach out to Zoé Hendrickson.

## Appendix 1

**Table 3:** Analytical framework approach

Study Sub-Group	Mothers	Health Extension Workers	Other Health Actors
Thematic Areas	• Background (Home, Livelihood, Education, Social Network) • Relationship/Marriage (History, relationship, division of work/labor, communication/conflict) • Motherhood experience (Mothering and household labor, childcare support, relationship with baby) • Health/well-being (Physical health, mental health, care-seeking) • Family planning (Fertility desires, FP)	• Roles and duties (Activities/responsibilities, clients/patients, workplace, postpartum care, integration of mental health) • Family planning (Postpartum FP training, postpartum FP approach, perceptions of postpartum FP among mothers) • Mental health (Training, perceptions of mental health among mothers, approaches for addressing mental health, mental health & careseeking) • Intervention experience (Mothers Time Training, Intervention Structure, Intervention materials, Sessions 1/2/3, Homework)	• Roles and duties (Activities/responsibilities, clients/patients, workplace, postpartum care) • Family planning (Postpartum FP training, postpartum FP approach, perceptions of postpartum FP among mothers) • Mental health (Training, perceptions of mental health among mothers, approaches for addressing mental health) • Intervention ideas (Interest in Mental Health, ideas, materials)

## Footnotes

1 The World Health Organization defines women with unmet need as “those who are fecund and sexually active but are not using any method of contraception and report not wanting any more children or wanting to delay the next child” [1]. Though specific definitions for unmet need vary, these measures help researchers and providers to understand the relationship between people’s fertility intentions and their contraceptive practices [1]. Throughout this paper, we cite several studies who use the term “unmet need” for family planning. Note that these papers may use slightly different definitions.

2 We also use definitions for “depression” and “anxiety” from the World Health Organization and the American Psychological Association, respectively. Depression is “characterized by persistent sadness and a lack of interest or pleasure in previously rewarding or enjoyable activities… it can also disturb sleep and appetite” [7]. Anxiety is characterized by “excessive feelings of tension, worried thoughts and physical changes like increased blood pressure” [8].

3 Researchers and practitioners define the timing of the postpartum period differently; we use the term to refer to the first year after birth.

4 CBT is based on a simple premise, which is that an individuals’ thinking and beliefs influence their feelings and emotional state, as well as their behavior, and that learning to notice and challenge beliefs can provide relief to mental health conditions like depression and anxiety [25]. CBT can be delivered through several formats, including individual sessions, group therapy, or mobile applications. CBT practitioners employ a range of techniques, including role-playing, mood logs, and activity scheduling [26].

5 Ethiopia’s National Guidelines for Family Planning Services describe some of the recent progress in expanding access to family planning, along with ongoing challenges and need [31]. Total unmet need for family planning among married women has declined in Ethiopia in recent decades, from 37% in 2000 to 22% in 2016 [33]. Alongside these gains, the maternal mortality and infant mortality ratios have declined by 53% and 50%, respectively [32]. Yet, key segments of the population continue to have unmet need. Postpartum FP uptake is low, with data from 2016 showing that only 26% of women use a method in the first year postpartum [34]. Ethiopia’s National Guidelines for Family Planning Services note the need for FP services for postpartum women [32].

6 Ethiopia’s National Mental Health Strategic Plan for 2020 - 2025 outlines the prevalence of various mental health conditions and sets strategy and goals for strengthening mental health on many levels, including at the community level [35]. The strategy notes that challenges in maternal mental health impact not just mothers, but their families and communities, and outlines plans to strengthen detection and response to maternal depression and anxiety. A recent systematic review and meta-analysis estimated the total prevalence of postpartum depression in Ethiopia to be at about 22%, in line with global estimates of the disorder [36].

7 Note that these screeners are not diagnostic tools. Mental health professionals can provide diagnoses for mental health disorders.

8 Data collection guides and training documents are available upon request.

## References

[1] World Health Organization. The Global Health Observatory, Unmet need for family planning (%). World Health Organization, 2023. Available from: https://www.who.int/data/gho/indicator-metadata-registry/imr-details/3414#:~:text=Unmet%20need%20is%20a%20rights,to%20delay%20or%20limit%20births.

[2] Yaya S, Uthman OA, Ekholuenetale M, Bishwajit G, Adjiwanou V. Effects of birth spacing on adverse childhood health outcomes: evidence from 34 countries in sub-Saharan Africa. J Matern Fetal Neonatal Med. 2020;33(20):3501–8. 30696314 10.1080/14767058.2019.1576623

[3] Conde-Agudelo A, Belizán JM. Maternal morbidity and mortality associated with interpregnancy interval: cross sectional study. BMJ. 2000;321(7271):1255–9. 11082085 10.1136/bmj.321.7271.1255 PMC27528

[4] Ross JA, Winfrey WL. Contraceptive use, intention to use and unmet n∙eed during the extended postpartum period. Int Fam Plan Perspect. 2001;1:20–7.

[5] Dev R, Kohler P, Feder M, Unger JA, Woods NF, Drake AL. A systematic review and meta-analysis of postpartum contraceptive use among women in low-and middle-income countries. Reprod Health. 2019;16:1–7. 31665032 10.1186/s12978-019-0824-4 PMC6819406

[6] Gahungu J, Vahdaninia M, Regmi PR. The unmet needs for modern family planning methods among postpartum women in Sub-Saharan Africa: a systematic review of the literature. Reprod Health. 2021;18:1–5. 33568180 10.1186/s12978-021-01089-9 PMC7877117

[7] World Health Organization. Depression. Geneva : World Health Organization; 2022. Available from: https://www.who.int/health-topics/depression#tab=tab_1.

[8] American Psychological Association. Anxiety. Washington: American Psychological Association; 2022. Available from: https://www.apa.org/topics/anxiety.

[9] Fisher J, Mello MC, Patel V, Rahman A, Tran T, Holton S, Holmes W. Prevalence and determinants of common perinatal mental disorders in women in low-and lower-middle-income countries: a systematic review. Bull World Health Organ. 2012;90:139–49. 10.2471/BLT.11.091850 PMC3302553 22423165

[10] Slomian J, Honvo G, Emonts P, Reginster JY, Bruyère O. Consequences of maternal postpartum depression: A systematic review of maternal and infant outcomes. Womens Health. 2019;15:1745506519844044. 10.1177/1745506519844044 PMC6492376 31035856

[11] McNab SE, Dryer SL, Fitzgerald L, Gomez P, Bhatti AM, Kenyi E, Somji A, Khadka N, Stalls S. The silent burden: a landscape analysis of common perinatal mental disorders in low-and middle-income countries. BMC Pregnancy Childbirth. 2022;22(1):1–4. 35443652 10.1186/s12884-022-04589-z PMC9019797

[12] Betancourt TS, Brennan RT, Vinck P, VanderWeele TJ, Spencer-Walters D, Jeong J, Akinsulure-Smith AM, Pham P. Associations between mental health and Ebola-related health behaviors: a regionally representative cross-sectional survey in post-conflict Sierra Leone. PLoS Med. 2016;13(8):e1002073. 27505186 10.1371/journal.pmed.1002073 PMC4978463

[13] Jenkins R, Othieno C, Ongeri L, Ongecha M, Sifuna P, Omollo R, Ogutu B. Malaria and mental disorder: a population study in an area endemic for malaria in Kenya. World Psychiatry. 2017;16(3):324. 28941104 10.1002/wps.20473 PMC5608856

[14] Nel A, Kagee A. Common mental health problems and antiretroviral therapy adherence. AIDS Care. 2011;23(11):1360–5. 22022846 10.1080/09540121.2011.565025

[15] Hummel AD, Ronen K, Bhat A, Wandika B, Choo EM, Osborn L, Batra M, Kinuthia J, Kumar M, Unger JA. Perinatal depression and its impact on infant outcomes and maternal-nurse SMS communication in a cohort of Kenyan women. BMC Pregnancy Childbirth. 2022;22(1):1–6. 36138357 10.1186/s12884-022-05039-6 PMC9494796

[16] Garbers S, Correa N, Tobier N, Blust S, Chiasson MA. Association between symptoms of depression and contraceptive method choices among low-income women at urban reproductive health centers. Matern Child Health J. 2010;14:102–9. 19067135 10.1007/s10995-008-0437-y

[17] Hall KS, Moreau C, Trussell J, Barber J. Young women’s consistency of contraceptive use—does depression or stress matter? Contraception. 2013;88(5):641–9. 23850075 10.1016/j.contraception.2013.06.003 PMC3796023

[18] Hall KS, White KO, Rickert VI, Reame N, Westhoff C. Influence of depressed mood and psychological stress symptoms on perceived oral contraceptive side effects and discontinuation in young minority women. Contraception. 2012;86(5):518–25. 22673038 10.1016/j.contraception.2012.04.010 PMC3440521

[19] Schonewille NN, Rijkers N, Berenschot A, Lijmer JG, van den Heuvel OA, Broekman BF. Psychiatric vulnerability and the risk for unintended pregnancies, a systematic review and meta-analysis. BMC Pregnancy Childbirth. 2022;22(1):153. 35216573 10.1186/s12884-022-04452-1 PMC8876535

[20] Catalao R, Medhin G, Alem A, Dewey M, Prince M, Hanlon C. Mental health impact on the unmet need for family planning and fertility rate in rural Ethiopia: a population-based cohort study. Epidemiology and Psychiatric Sciences. 2020;29:e160. 32807254 10.1017/S2045796020000736 PMC7443804

[21] Haider TL, Sharma M. Barriers to family planning and contraception uptake in sub-Saharan Africa: a systematic review. Int Q Community Health Educ. 2013;33(4):403–13. 10.2190/IQ.33.4.g 24044930

[22] Fricchione GL, Borba CPC, Alem A, Shibre T, Carney JR, Henderson DC. Capacity Building in Global Mental Health: Professional Training. Harv Rev Psychiatry. 2012;20(1):47–57. 10.3109/10673229.2012.655211. 22335182 10.3109/10673229.2012.655211 PMC3335114

[23] World Health Organization. Investing in mental health: evidence for action. Geneva: World Health Organization; 2013. Available from: https://iris.who.int/bitstream/handle/10665/87232/9789241564618_eng.pdf.

[24] David D, Cristea I, Hofmann SG. Why Cognitive Behavioral Therapy Is the Current Gold Standard of Psychotherapy. Front Psychiatry. 2018;9:4. 10.3389/fpsyt.2018.00004 PMC5797481 29434552

[25] Beck AT. Cognitive therapy: Nature and relation to behavior therapy–republished article. Behav Ther. 2016;47(6):776–84. 27993332 10.1016/j.beth.2016.11.003

[26] Lam DC. A brief overview of CBT Techniques. In: Freeman MS and Freeman A, editors. Cognitive behavior therapy in nursing practice. New York, Springer Publishing Company, Inc, 2004.

[27] Murray LK, Skavenski S, Kane JC, Mayeya J, Dorsey S, Cohen JA, Michalopoulos LT, Imasiku M, Bolton PA. Effectiveness of trauma-focused cognitive behavioral therapy among trauma-affected children in Lusaka, Zambia: a randomized clinical trial. JAMA Pediatr. 2015;169(8):761–9. 26111066 10.1001/jamapediatrics.2015.0580 PMC9067900

[28] Senyonyi RM, Underwood LA, Suarez E, Musisi S, Grande TL. Cognitive behavioral therapy group intervention for HIV transmission risk behavior in perinatally infected adolescents. Health. 2012;4(12):1334–45.

[29] Sikander S, Maselko J, Zafar S, Haq Z, Ahmad I, Ahmad M, Hafeez A, Rahman A. Cognitive-behavioral counseling for exclusive breastfeeding in rural pediatrics: a cluster RCT. Pediatrics. 2015;135(2):e424–31. 25583916 10.1542/peds.2014-1628

[30] Rahman A, Malik A, Sikander S, Roberts C, Creed F. Cognitive behaviour therapy-based intervention by community health workers for mothers with depression and their infants in rural Pakistan: a cluster-randomised controlled trial. The Lancet. 2008;372(9642):902–9. 10.1016/S0140-6736(08)61400-2 PMC2603063 18790313

[31] World Health Organization. Thinking Healthy: A Manual for Psychosocial Management of Perinatal Depression (WHO generic field-trial version 1.0). Geneva: WHO; 2015.

[32] Federal Democratic Republic of Ethiopia Ministry of Health. National Guideline for Family Planning Services in Ethiopia. Addis Ababa: Federal Democratic Republic of Ethiopia Ministry of Health; 2020.

[33] The DHS Program. STATcompiler. Available from : https://www.statcompiler.com/en/.

[34] Track20. Opportunities for Family Planning Programming in the Postpartum Period in Ethiopia. Track20, 2016. Available from: https://www.track20.org/download/pdf/PPFP%20Opportunity%20Briefs/english/Ethiopia%20PPFP%20Opportunity%20Brief%202.pdf.

[35] Ministry of Health – Ethiopia. National Mental Health Strategic Plan. Addis Ababa, Ministry of Health – Ethiopia, 2020. Available from: https://www.mhinnovation.net/resources/national-mental-health-strategy-ethiopia#:~:text=A%20critical%20milestone%20for%20Ethiopia's,a%20wide%20range%20of%20stakeholders.

[36] Zeleke TA, Getinet W, Tadesse Tessema Z, Gebeyehu K. Prevalence and associated factors of post-partum depression in Ethiopia. A systematic review and meta-analysis. PloS one. 2021;16(2):e0247005. 10.1371/journal.pone.0247005 PMC7894900 33606768

[37] Federal Democratic Republic of Ethiopia Population Census Commission. Summary and Statistical Report of the 2007 Population and Housing Census. Addis Ababa, United Nations Population Fund, 2008. Available from: https://www.ethiopianreview.com/pdf/001/Cen2007_firstdraft(1).pdf.

[38] Alemu Y, Dessie H, Birara M. Risk factors of mortality among children under age five in Awi Zone, northwest Ethiopia. PLoS ONE. 2022;17(10):e0275659. 36197924 10.1371/journal.pone.0275659 PMC9534439

[39] Mitiku HD, Lemma MW, Chekole YB, Chekole YT. Hierarchical Analysis of Contraceptive Compliance Among Rural Reproductive Age Group Women in Awi Zone. Northwest Ethiopia Patient preference and adherence. 2022;24:1279–93. 10.2147/PPA.S366097 PMC9147396 35637685

[40] Wang H, Tesfaye R, Ramana GN, Chekagn CT. Ethiopia health extension program: an institutionalized community approach for universal health coverage. World Bank Publications; 2016.

[41] Mothers and Babies. Resources. Chicago: Mothers and Babies Program; 2000.

[42] Kroenke K, Spitzer RL, Williams JB. The PHQ-9: validity of a brief depression severity measure. J Gen Intern Med. 2001;16(9):606–13. 11556941 10.1046/j.1525-1497.2001.016009606.x PMC1495268

[43] Williams N. The GAD-7 questionnaire. Occupational Med. 2014;64(3):224-.

[44] Hanlon C, Medhin G, Selamu M, Breuer E, Worku B, Hailemariam M, Lund C, Prince M, Fekadu A. Validity of brief screening questionnaires to detect depression in primary care in Ethiopia. J Affect Disord. 2015;1(186):32–9. 10.1016/j.jad.2015.07.015 26226431

[45] Bitew T, Keynejad R, Myers B, Honikman S, Medhin G, Girma F, Howard L, Sorsdahl K, Hanlon C. Brief problem-solving therapy for antenatal depressive symptoms in primary care in rural Ethiopia: protocol for a randomised, controlled feasibility trial. Pilot and Feasibility Studies. 2021;7(1):1–3.> 33514447 10.1186/s40814-021-00773-8 PMC7846490

[46] Lundgren R, Burgess S, Chantelois H, Oregede S, Kerner B, Kågesten AE. Processing gender: lived experiences of reproducing and transforming gender norms over the life course of young people in Northern Uganda. Cult Health Sex. 2019;21(4):387–403. 29882476 10.1080/13691058.2018.1471160

[47] Igras S, Burgess S, Chantelois-Kashal H, Diakité M, Giuffrida M, Lundgren R. Pathways to modern family planning: a longitudinal study on social influence among men and women in Benin. Stud Fam Plann. 2021;52(1):59–76. 33559166 10.1111/sifp.12145 PMC8048892

[48] Yitbarek K, Birhanu Z, Tucho GT, Anand S, Agenagnew L, Ahmed G, Getnet M, Tesfaye Y. Barriers and facilitators for implementing mental health services into the Ethiopian Health Extension Program: a qualitative study. Risk Management and Healthcare Policy. 2021;19:1199–210. 10.2147/RMHP.S298190 PMC7989539 33776497

[49] Astale T, Abebe T, Mitike G. Workload and emerging challenges of community health workers in low-and middle-income countries: A mixed-methods systematic review. PLoS ONE. 2023;18(3):e0282717. 36913362 10.1371/journal.pone.0282717 PMC10010520

[50] Costenbader E, Lenzi R, Hershow RB, Ashburn K, McCarraher DR. Measurement of social norms affecting modern contraceptive use: A literature review. Stud Fam Plann. 2017;48(4):377–89. 29165824 10.1111/sifp.12040

[51] Kim TY, Igras S, Barker KM, Diakité M, Lundgren RI. The power of women’s and men’s Social Networks to catalyse normative and behavioural change: evaluation of an intervention addressing Unmet need for Family Planning in Benin. BMC Public Health. 2022;22(1):1–4. 35392862 10.1186/s12889-022-12681-4 PMC8988370

[52] Hutchinson P, Schoop J, Andrinopoulos K, Do M. Assessment report for the Hewlett Foundation’s strategy to apply behavioral economics to improve family planning and reproductive health service delivery. New Orleans (LA): Tulane University School of Public Health and Tropical Medicine; 2018.

[53] Kakuma R, Minas H, Van Ginneken N, Dal Poz MR, Desiraju K, Morris JE, Saxena S, Scheffler RM. Human resources for mental health care: current situation and strategies for action. The Lancet. 2011Nov 5;378(9803):1654–63. 10.1016/S0140-6736(11)61093-3 22008420

[54] Jaskiewicz W, Tulenko K. Increasing community health worker productivity and effectiveness: a review of the influence of the work environment. Hum Resour Health. 2012;10(1):1–9. 23017131 10.1186/1478-4491-10-38 PMC3472248

[55] Senderowicz L. “I was obligated to accept”: a qualitative exploration of contraceptive coercion. Soc Sci Med. 2019Oct;1(239):112531. 10.1016/j.socscimed.2019.112531 31513932

